# Posterior Reversible Encephalopathy Syndrome Presenting as Opsoclonus-Myoclonus

**DOI:** 10.3109/01658107.2012.667186

**Published:** 2012-07-30

**Authors:** Torrey Boland, Jamie Strause, Myra Hu, Dolores Santamaria, Tsao-Wei Liang, Daniel Kremens, Robert Sergott, Michael Moussouttas

**Affiliations:** ^a^ Department of Neurology, Thomas Jefferson Medical Center,Philadelphia, Pennsylvania, USA; ^b^ Advocare Neurology, Virtua Memorial Hospital,Lumberton, New Jersey, USA; ^c^ Movement Disorders Division, Thomas Jefferson Medical Center,Philadelphia, Pennsylvania, USA; ^d^ Neuro-Ophthalmology Service, Thomas Jefferson Medical Center,Philadelphia, Pennsylvania, USA; ^e^ Cerebrovascular and Neurocritical Care Division, Thomas Jefferson Medical Center,Philadelphia, Pennsylvania, USA

**Keywords:** Cerebellum, Dentate, Myoclonus, Opsoclonus, PRES

## Abstract

Opsoclonus-myoclonus may be caused by various neurological conditions and toxic-metabolic states, but typically occurs as a parainfectious or paraneoplastic manifestation. The development of opsoclonus-myoclonus has been variably attributed to lesions in the pons or cerebellum. Herein the authors describe a case of opsoclonus-myoclonus due to posterior reversible encephalopathy syndrome in which magnetic resonance imaging revealed lesions in the region of the cerebellar dentate nuclei. Clinical and radiological resolution of the opsoclonus-myoclonus and of the posterior reversible encephalopathy syndrome followed antihypertensive therapy.

## INTRODUCTION

Posterior reversible encephalopathy syndrome (PRES) typically presents with headache, visual loss, seizures, and confusion.^1^ It is frequently associated with hypertension and may occur in the presence of organ failure, immune diseases, and immunosuppresent or antineoplastic medications.^1^ Opsoclonus-myoclonus (OM) has traditionally been described as a paraneoplastic or as a parainfectious entity, or in conjunction with specific toxic-metabolic conditions.^2^ Herein we report a case of PRES presenting as OM; we are not aware of any previous report of such a case.

### CASE REPORT

A 60-year-old man with a history of hypertension, hyperlipidaemia, and idiopathic peripheral neuropathy presented to a local hospital with a 1-week history of visual changes and increasing confusion. Family members had noticed transient episodes of involuntary twitching in his limbs. In the emergency room, mean arterial pressure (MAP) was 150 mm Hg (controlled baseline ~120/70) and the patient was confused and agitated. Admission serology revealed acute renal insufficiency (creatinine [Cr] 3.5 mg/dL) and hepatic dysfunction (aspartate aminotransferase [AST] 1128 units/L, alanine aminotransferase [ALT] 1000 units/L), as well as rhabdomyolysis (creatine phosphokinase [CPK] 4415 units/L). The patient was noted to have pronounced periodic involuntary movements of his extremities, for which he was intubated and started on propofol for presumed status epilepticus. The patient was subsequently transferred to our institution for continuous electroencephalogram (EEG) monitoring.

**FIGURE 1:**
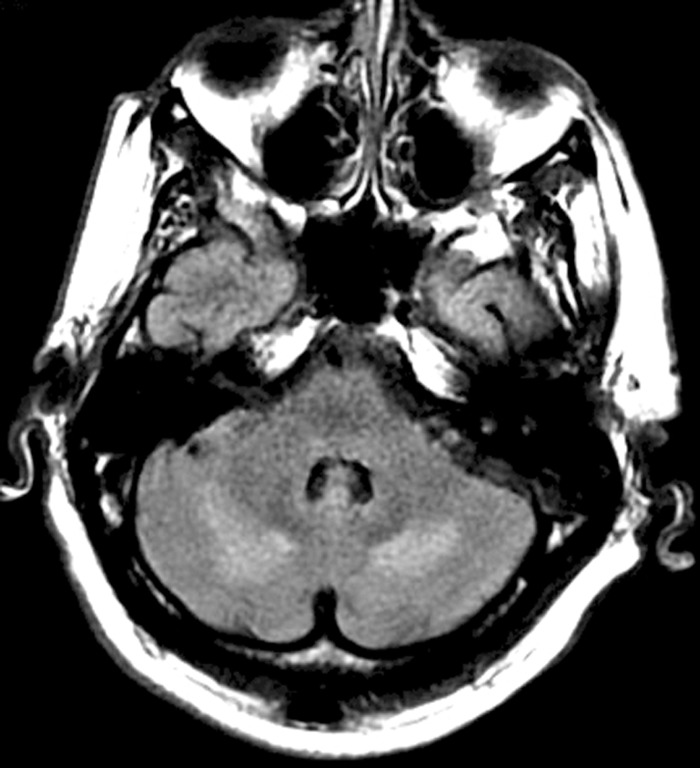
Initial fluid attenuated inversion recovery (FLAIR) MR sequence demonstrating hyperintense lesions adjacent to the cerebellar dentate nuclei bilaterally. No additional lesions were identified on this MR series.

**FIGURE 2:**
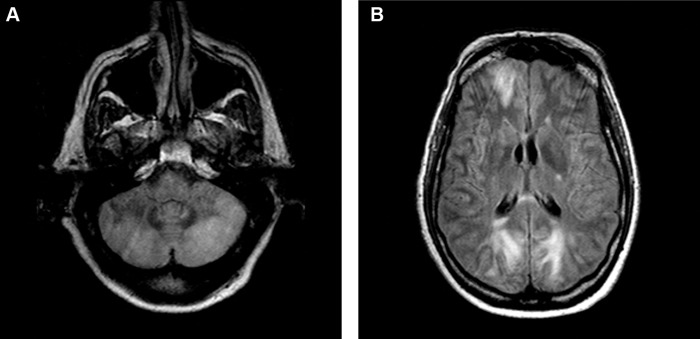
Repeat MR imaging (FLAIR sequence) demonstrating more extensive involvement of the cerebellum (A) and oedema in the subcortex of the frontal and occipital lobes (B) consistent with PRES.

Upon arrival at our institution, systemic pressure was 170/70 mm Hg. On neurological examination, the patient was agitated, nonverbal, unable to follow commands, and exhibited multifocal asynchronous and arrhythmic myoclonic movements of all extremities. Ophthalmic examination demonstrated repetitive, rapid, random, multidirectional, involuntary conjugate saccades of irregular amplitude and frequency without an intersaccadic interval that decreased somewhat with eye closure. Review of the magnetic resonance (MR) imaging carried out at the transferring hospital demonstrated T2 signal hyperintensities in the areas of the cerebellar dentate nuclei bilaterally (Figure 1). Repeat MR imaging at our institution (performed 3 days after initial imaging) revealed subcortical hyperintensities in the frontal and bilateral posterior hemispheres, as well as in the cerebellum bilaterally, indicative of oedema and consistent with PRES (Figure 2).

EEG monitoring during active myoclonus demonstrated only generalised slowing. Cerebrospinal fluid (CSF) examination revealed glucose 102 mg/dL, protein 124 mg/dL, 9 erythrocytes/μL, and 7 leukocytes/μL. CSF bacterial, tuberculous, and fungal stains and cultures were negative. Cytology was negative for neoplastic cells. CSF *Listeria*, Lyme, and syphilis serology was negative. Polymerase chain reaction (PCR) testing for herpes simplex 1 and 2, Epstein-Barr virus, cytomegalovirus, varicella-zoster, West Nile antibody, and JC virus were all negative. Plasma, sputum, and urine samples revealed normal leukocyte counts, and negative bacterial and fungal cultures. Full-body computed tomography (CT) with intravenous and oral contrast showed evidence of neither a primary nor metastatic neoplasm.

Serum anti-nuclear antibody (ANA), anti-double-stranded DNA, anti-nuclear cytoplasmic antibody (ANCA) panel, anti-thyroid antibodies, and antiphospholipid assays (anti-cardiolipin, lupus anticoagulant, β_2_-glycoprotein) were negative. CSF paraneoplastic panel ANNA-1 (anti-Hu), ANNA-2 (anti-Ri), ANNA-3, PCA-1 (anti-Yo), PCA-2, PCA-Tr, AGNA-1, CRMP-5, anti-CAR, and amphyphysin antibodies was unrevealing. Tests for various forms of porphyria and for mitochondrial diseases were normal. Serum and urine protein electrophoresis studies were consistent with monoclonal gammopathy of uncertain significance (MGUS), and bone marrow biopsy, flow cytometry and chromosome analysis excluded the presence of myelomatous disease. Urine toxicology was negative.

Following review of the MR imaging studies a diagnosis of PRES was made in the context of a hypertensive crisis. The patient was treated with intravenous nicardipine to maintain a MAP of <100 mm Hg for the first 24 h, and subsequently a MAP of <90 mm Hg. After 48 h of successful hypertensive management, mental status markedly improved and the OM completely resolved. The CPK, creatinine, and transaminase levels rapidly returned to normal. Repeat MR imaging (performed 9 days after initial imaging and 6 days following second scan) demonstrated partial resolution of the subcortical oedema. The patient was discharged to an acute rehabilitation facility, and had no subsequent hypertensive crises. MR imaging (performed several months later) revealed complete resolution apart from a minor focus of residual subcortical hyperintensity in the left posterior temporal lobe. One year following admission, a repeat evaluation for malignancy remains unrevealing.

## DISCUSSION

Movement disorders associated with PRES are exceptionally rare, with only a single report of dystonia observed among several recent case series.^3^ Individual reports of involuntary ocular movements associated with PRES include one case of oculogyric crisis and a single case of isolated opsoclonus, each following organ transplantation and cyclosporine treatment in paediatric patients.^4,5^ In contrast to the latter, our patient developed the complete OM syndrome, did not receive organ transplantation or immunosuppressant therapy, and revealed potentially causative lesions in the posterior fossa on cerebral imaging.

In adults, OM typically presents as a paraneoplastic syndrome, usually related to lung or breast carcinoma (at times related to an ANNA-2 antibody), or as a parainfectious syndrome in the setting of acute encephalitis, but may also rarely result from intracranial processes and from various toxins and metabolic conditions.^2^ OM is characterised by involuntary, conjugate, rapid, multidimensional, recurrent saccades of varying amplitude and direction, and by spontaneous multifocal asynchronous and arrhythmic jerking of the torso and limbs.^2^ Cerebral imaging studies are usually unrevealing,^6^ yet some investigations have identified possible explanatory radiological and physiological anomalies.

Various candidate neuroanatomical structures have been implicated in the pathophysiology of OM. Lesions of the pons involving damage to the omnipause cells, which normally inhibit the burst neurons responsible for generating the saccadic impulse,^7^ or cerebellar lesions interrupting normal vermian inhibition of the fastigial nucleus, which regulates vestibular nuclear tone to burst neurons,^8–11^ have been suspected. Impairment of the dentatothalamocortical pathway has also been suggested,^12^ and supported by studies that have identified histological and biochemical alterations in the dentate nuclei.^13,14^ Concurrent pontine and cerebellar involvement has also been proposed.^15^

In our patient, hypertensive exacerbation in conjunction with organ dysfunction, and possibly also altered immune function (MGUS), may have contributed to the development of PRES. Whereas the acute renal insufficiency may have been caused by the crisis itself, renal and hepatic dysfunction may also have been due to the systemic endothelial damage and organ dysfunction that may occur with PRES.^1^ The mildly elevated CSF protein and leukocyte levels were also consistent with prior observations in patients with PRES.^16^ Considering that no underlying malignancy or paraneoplastic antibody was identified, that an extensive infectious disease evaluation was unrevealing, and that clinical and radiological resolution occurred with antihypertensive therapy, we believe that OM in this case was due to PRES.

We cannot entirely exclude a role for metabolic derangement in contributing to myoclonus in our patient, yet believe that the extent of organ dysfunction was relatively minor. In addition, myoclonus usually develops in chronic renal conditions more so than with acute injury, and hepatic disorders typically result in asterixis instead of myoclonus.^17^ Most importantly, renal and hepatic failure have never been associated with opsoclonus or the OM syndrome.^2,6,18^ Finally, despite the finding of cerebellar lesions, we cannot entirely exclude involvement of additional posterior fossa structures below the resolution of MR imaging in the localization of OM.

## CONCLUSION

In conclusion, we present a unique case of PRES manifesting as OM, in which cerebral imaging revealed lesions in the region of the dentate nuclei of the cerebellum. Controversy exists as to the exact neuroanatomic localization and mechanism of OM, but our case is consistent with several current theories regarding a possible cerebellar origin for this movement disorder. We recommend that PRES be considered in the differential diagnosis for patients presenting with OM syndrome.
